# The Relationship between Anger Expression and Performance Score in Parents and Coaches: The Mediating Role of Self-Efficacy and Assertiveness

**DOI:** 10.3390/ijerph20075372

**Published:** 2023-04-03

**Authors:** Donatella Di Corrado, Elisabetta Sagone, Andrea Buscemi, Marinella Coco

**Affiliations:** 1 Department of Sport Sciences, Kore University, Cittadella Universitaria, 94100 Enna, Italy; 2 Department of Education Sciences, University of Catania, 95124 Catania, Italy; 3 Study Center of Italian Osteopathy and Horus Social Cooperative, 95100 Catania, Italy

**Keywords:** performance, parents, coaching, children, physical activity

## Abstract

Background: Youth sport provides regular physical activity for millions of children. It is a global system, which includes coaches, referees, athletes, and parent spectators; consequently, the behavior of each member can influence the experiences of others. This study aimed to investigate the mediating role of self-efficacy and assertiveness in the relationship between the degree of anger expression and the performance children’s score in a group of parents and coaches. Methods: 200 parents (96 fathers, 104 mothers) and 200 coaches (119 males, 81 females) responded to the General Self-efficacy Scale, The Scale for Interpersonal Behavior, and the State–Trait Anger Expression Inventory-2; an indicator of performance was asked of each coach. The age range of parents was 28–59 years (M = 41.39, SD = 7.07), while that of coaches was 27–43 years (M = 35.91, SD = 3.35). Results: Results revealed that self-efficacy and assertiveness were the direct and indirect predictors of performance score. Conclusions: The study provided an understanding of the nature and magnitude of this profoundly interpersonal experience. Future studies may advance relevant education programs and effective interventions aimed at reducing angry expressions and lack of communication.

## 1. Introduction

There are many positive benefits for children and adolescents who perform physical activity out of school hours. Sport participation is related with positive emotional well-being, and young people reported a high quality of life, social and emotional functioning, and correct development [1].

Recent systematic reviews suggested that children and adolescents with a high frequency of physical activity and exercise promote well-being and reduce risk of chronic diseases, such as obesity [2]. Through sports activities, young people can also improve their self-esteem and self-efficacy, learn about cooperation, and control aggressiveness [3].

Physical activity is described as a corporal movement that increases energy expenditure relative to rest, and involves sports, conditioning, and other activities to improve body composition, muscle power, cardio-respiratory effects, and balance [4].

During children’s and adolescence’s sports practice, three significant social agents are implicated: coaches, parents, and peers. From different theories and research, adult figures (e.g., program staff: teachers, coaches, and counselors) assumed a significant source of support for youth physical health outcomes and biopsychosocial development [5,6].

Fenton et al. [7] found that as coaches promote an empowering motivational climate in sport, youth have more fun, motivation, participation, and daily physical activity. In addition, parents are active participants in sports performance, investing an increasing percentage of necessary resources, equipment, time for structured and unstructured play, and emotional support. This is significant because parent involvement (such as communication, encouragement, and cohesion) improves youngsters’ outcomes (e.g., enjoyment, autonomy, and motivation) [8].

It is important to produce positive sports situations in which young athletes enjoy their experiences. In this regard, coaches are also considered to have a great influence on their lives, providing and creating a healthy and educational sports environment. Coaches can encourage youth to take up more challenging activities as well as simplify a pleasant experience for young athletes. For these reasons, coaches have an important educational and socialization role, and parents also play an essential motivating role [9].

Actually, sport is an activity that is very popular with parents, especially in terms of the success of the child. In fact, in sport, the success of the child is both visible and measurable, and too often parents claim sports skills, monitoring and following the athletic abilities of their children [10]. It is reasonable that parents’ interest in their children’s sports activities depend on their degree of participation in the sport and on parents’ presence of experience playing the same sports as their children [11].

It is necessary to address strategies of parental behaviors on physical activity promotion to foster a positive and healthy youth experience. Parents who are perceived to be effective in relation to the performance of physical exercise are able to motivate their child more to play sports and to maintain it over time [12].

Within physical activity promotion research, perceived self-efficacy has been defined as people’s beliefs in specific capabilities to execute the courses of action required to achieve a given outcome, to be successful in a task, and to exercise control over its functioning [13]. In this regard, choice of physical activity, the amount of time, and the level of effort are influenced from a strong sense of self-efficacy [14]. Individual’s beliefs about their ability can be developed from different sources: past performance activities, vicarious experiences, social persuasion, and physiological conditions. Research by Swensen and colleagues highlighted that perceived parental support can play an essential role for the development of self-confidence and abilities, promoting higher self-efficacy [15]. Several studies have theorized that another powerful factor in self-efficacy development was the athlete–coach relationship [16,17]. In fact, coaches promoted athlete self-efficacy development through inspiring positive self-talk, kindly offering praise, and modeling self-confidence themselves [18]. The concept of self-efficacy referring specifically to coaches and parents is very important in the sport settings.

First, self-efficacy is related to coaching of one’s capability to realize desired athletic outcome in players. A coach’s efficacy has been recognized as an important variable to predict athlete satisfaction, to know what athletes feel, and to better understand their psyche. According to the literature, more efficacious coaches had an increased perception of athlete skills and a higher level of coaching education [19,20]. Instead, parental self-efficacy has been designed as the expectation parents hold about their ability to execute effective educational practices. Parents with higher level of self-efficacy tend to use more encouraging parenting (e.g., warmness, positive affect, sensitivity, and constancy) than parents with a low self-efficacy, who use more forced and severe parenting [21,22]. Then, during the performance of young people’s sports, satisfactory parent/coach relationship assumes a decisive function and is related to better fun and motivation to continue [23]. However, various researchers have shown a difficult and unclear communication between parents and coaches of their children’s sports team. It negatively affects their relationships and the sports environment and often becomes the reason for the interruption of sport on the part of young people [24,25].

Parents and coaches need appropriate communication skills and assertiveness skills as well as encouragement to become actively involved in the youth development [26]. Assertiveness is regarded as a personality trait, and it is linked to the temperament. It represents the ability to properly express ideas, emotional states, and bounds, while respecting others’ rights, preserving a positive effect on the other, and considering the possible consequences of the expression use [27]. Therefore, the establishment of a positive and good relationship with coaches and/or parents is seen as a predictor of competitive performances. Unfortunately, many children participate in youth sport while parents scream or argue with coaches or other spectators in the background. Of course, watching a child participate in a youth sport performance is primarily an emotional, rather than a rational, experience for many parents [28]. Omli and LaVoi [29] found that the most difficult behaviors appear to be motivated by emotion of anger. The theoretical anger construct was defined by the existence of a verbal, non-verbal, or physical conflict between two or more persons without directly including a spectator [30]. In this regard, the inappropriate behaviors of parents or coaches can be considered as a potential source of stress for youth sport participants. Likewise, a coach who seems calm and confident will have a different effect on the youth than a coach who seems nervous or angry. Maintaining emotional control under situations of intense pressure is an important requirement of all competitive sport and involves the influence of self-efficacy and assertiveness [31].

Presently, coaches’ and parents’ influence have a growing popularity in the young sporting world. However, given the limited research, the purpose was to investigate the mediating role of self-efficacy and assertiveness in the relationship between the degree of anger expression and the performance children’s score in a group of parents and coaches. More specifically, we hypothesized the following:

**Hypothesis** **1** **(H1).** *Self-efficacy would be negatively associated with anger expression and general assertiveness distress.*

**Hypothesis** **2** **(H2).** *Self-efficacy and assertive behavior mediate the relationship between anger expression and performance score.*

## 2. Materials and Methods

### 2.1. Participants

An a priori power analysis was run with G*Power [32]. This analysis found a multiple regression analysis with 12 predictors, an alpha level of 0.05 (two-tailed), a power of 0.80 to detect a small to medium effect size of ρ^2^ = 0.06, and a required total sample size of *n* = 105. Therefore, a total of *n* = 400 participants were recruited to take part in the research. The sample consisted of 200 parents (96 fathers, 104 mothers) and 200 coaches (119 males, 81 females) of different sports, recruited from Public Schools and Sport Centers in Catania and Province, respectively. The age range of parents was 28–59 years (M = 41.39, SD = 7.07), while that of coaches was 27–43 years (M = 35.91, SD = 3.35). The age range of coaching activity was 10–32 years. Inclusion criteria for coaches were: (a) they were currently coaching athletes aged between 8 and 14 years old; (b) they were coaches of an individual or a collective sport; and (c) they could provide informed consent. Inclusion criteria for parents were: (a) their children were aged between 8 and 14 years; (b) their children were currently competing in individual or a collective sport; and (c) they could provide informed consent. Participants were excluded if did not meet the inclusion criteria.

The children in this study were 200 injury-free athletes from different sports: swimming (19.5%), soccer (27.5%), tennis (11%), volleyball (15%), dance (16%), and artistic gymnastic (11%), between the ages of 8 and 13 years (M age = 10.50, SD = 1.73). They had a minimum of 5 years of training skill in the sport, and they were all associates of sport clubs. Their hours of exercise per week ranged from 3.0 to 21.5 h (M = 7.39, SD = 3.41).

### 2.2. Measurement Instruments

#### 2.2.1. General Self-Efficacy

The Italian version of the General Self Efficacy Scale [33,34] is a 10-item scale designed to assess optimistic self-beliefs to cope with demands in life and reach goals (e.g., “Even if someone opposes, I can find the means and strength to achieve what I desire”; “I am confident that I can cope adequately with unexpected events”). Response options range on a 5-point Likert format from (5) Completely agree to (1) Completely disagree. Higher scores indicate a stronger sense of self-efficacy. The internal reliability Cronbach’s alpha is 0.90. The Italian version provided evidence for reliability and validity with minor modifications to the original version. In the present sample, Cronbach’s α was 0.89.

#### 2.2.2. The Scale for Interpersonal Behavior (SIB)

The Italian version of Scale for Interpersonal Behavior [35] is a 50-item multidimensional measure of assertiveness. In particular, it assesses the probability of response (performance) and the degree of discomfort (felt anxiety/distress) associated with attempts at self-assertion in a variety of social situations. Four dimensions are measured: negative assertion, expression of and dealing with personal limitations, initiating assertiveness, and positive assertion. Response options range on a 5-point Likert format from (1) No discomfort and tension to (5) A lot of discomfort and tension. The SIB has good reliability and adequate construct validity. Ranging from 0.70 to 0.91, test–retest correlations were shown to be excellent, as were the internal consistency coefficients which ranged from 0.73 to 0.91. In addition to these four dimensions, General Assertiveness scores, for both distress and performance (item scores are summated across all 50 items), can be used as an indication of a person’s level of assertiveness across various situations and various types of assertive responses. We have used only these scores. In the present sample, internal consistency reliabilities for the distress and performance scales were all more than satisfactory and ranged from 0.78 to 0.87.

#### 2.2.3. State–Trait Anger Expression Inventory-2 (STAXI-2)

The STAXI [36] is a questionnaire consisting of 57 items that measure the experience of anger intended as a psychobiological emotional state. It comprises the state of anger marked by self-descriptive feelings of different intensities (15 items—e.g., “I am furious”; “I feel irritated”; “I feel angry”). Participants rate themselves on a 4-point frequency scale from (1) At all to (4) Really much. The trait anger measures the general disposition to perceive a great number of situations as annoying or frustrating with no reference to circumstances and without provocation (10 items—e.g., “I am quick-tempered”; “I have a fiery temper”). Participants rate themselves on a 4-point frequency scale from (1) Almost never to (4) Almost always. Finally, the last 32 items measure the experience of anger as an expression of the same (anger/out, anger/in, control/out, control/in item—e.g., “I lose my temper”; “I control my temper”; “I express my anger”; “I take a deep breath and relax”). Ratings are again on a 4-point frequency scale from (1) Almost never to (4) Almost always. The STAXI exhibits good reliability, with alpha coefficients ranging from 0.81 to 0.93. An index of anger expression can be derived to provide a summarizing measure of the expression and control of anger. In this study, we have used the Italian adaptation of STAXI-2 [37]. It has been shown to reflect the original factorial structure, with an acceptable internal consistency of the subscales and ranged from 0.71 to 0.89. The internal consistency for the current study is very good (α = 0.88).

#### 2.2.4. Performance Data

To obtain an indicator of performance attainment, we asked each coach to rate the performance of their players relative to others. The assessment was carried out using a 100-point scale [38], with raters being informed that “>60 was the passing grade”, thus <60 indicated low performance score and >60 indicated high performance score.

### 2.3. Procedure

All participants were examined separately in individual meetings lasting approximately 20 min. Coaches were tested in an isolated place near the training accommodations. Parents were tested in a separate location near the sport clubs at the end of the activities. The participant was required to answer as rapidly and as correctly as possible. During the test trials, no feedback was provided. Prior to the beginning of the study, ethical approval was granted from the first author’s university ethics committee. All the procedures were conducted in accordance with the ethics of the Declaration of Helsinki, and the University Enna Kore Internal Review Board for psychological research (UKE-IRBPSY-03.23.01) granted ethical permission. All participants were informed of the procedures of the study and the anonymity of their answers before providing their written consent to participate.

### 2.4. Statistical Analysis

The Kolmogorov–Smirnov test showed a normal distribution of the data. Data are expressed as means ± standard deviations (s) and the range. A Student’s *t*-test was used to detect the mean differences between coaches and parents. Additionally, Spearman’s rho correlation coefficient was used to determine the relationship between the selected variables. A mediation analysis was performed to study whether the relationship between anger expression (independent variable) and the performance children’s score (dependent variable) was mediated by self-efficacy and general assertiveness (distress and performance). To avoid biased parameter estimates, a multiple mediation model, which involves “simultaneous mediation by multiple variables” with Hayes’ (Hayes PROCESS version 4.0, Model 4–Ohio, MO, USA) computational tool [39] for SPSS was used in the current study. Hayes PROCESS can be used also when the dependent variable (Y) is binary, as in the present study. Statistical significance was set at *p* ≤ 0.05. Statistical analyses were processed using SPSS version 27.0 (IBM, Armonk, NY, USA).

## 3. Results

### 3.1. Group and Performance Score Differences

Table 1 shows the comparison between parents and coaches conducted through the Student’s *t*-test (*p* < 0.05).

As seen above, the general assertiveness distress was significantly higher in parents than in coaches. Moreover, parents had significantly lower levels of general assertiveness performance. The anger expression was significantly higher in parents than in coaches.

Table 2 shows the comparison between low-performance score and high-performance score conducted through the Student’s *t*-test (*p* < 0.05). The assessment was carried out using a global 0–100 scale, with raters being informed that “>60 was the passing grade”, thus <60 indicated low-performance score and >60 indicated high-performance score.

As seen above, self-efficacy and general assertiveness performance were significantly higher in high-performance score than in low score. Moreover, general assertiveness distress and anger expression dimension were significantly lower in high-performance score than in low-performance score.

### 3.2. Correlations

The relationships between the variables of the study were analyzed using Spearman’s rho correlation coefficient. Results showed that self-efficacy was significantly and negatively associated with scores for general assertiveness distress (*p* < 0.01; *r* = −0.620) and anger expression (*p* < 0.01; *r* = −0.552) and positively associated with general assertiveness performance (*p* < 0.01; *r* = 0.658). On the other hand, scores for the general assertiveness performance were significantly and negatively related to general assertiveness distress (*p* < 0.01; *r* = −0.664). Moreover, anger expression was significantly and positively associated with scores for general assertiveness distress (*p* < 0.01; *r* = 0.593) and negatively associated with general assertiveness performance (*p* < 0.01; *r* = −0.549).

### 3.3. Mediation Analysis

On the basis of the correlation results, a mediation analysis was executed by entering the scores for self-efficacy and general assertiveness (distress and performance) as mediators in the anger expression and the performance children’s score relationship. Specifically, we ran two mediation models, considering the two different groups of participants (parents and coaches). In all models, anger expression was the independent variable, while self-efficacy and general assertiveness (distress and performance) were mediators, and performance children’s score was the dependent variable (binary outcome).

**Figure 1:**
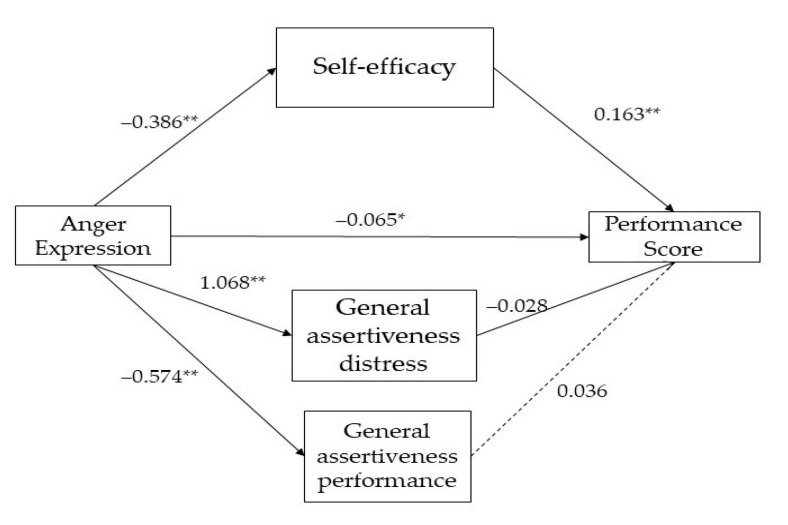
Parallel-multiple mediation of self-efficacy and general assertiveness (distress and performance) between anger expression and performance children’s score (group of parents). Note: * *p* < 0.01; ** *p* < 0.001. Dashed lines indicate non-significant effects.

**Figure 2:**
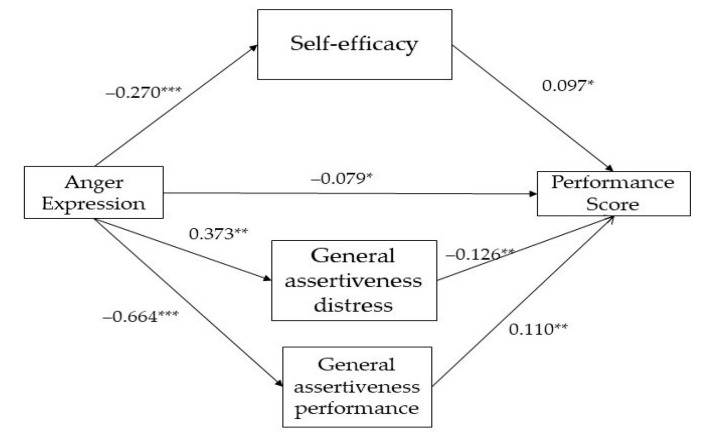
Parallel-multiple mediation of self-efficacy and general assertiveness (distress and performance) between anger expression and performance children’s score (group of coaches). Note: * *p* < 0.05; ** *p* < 0.01; *** *p* < 0.001. Dashed lines indicate non-significant effects.

The results for the two mediation models are presented in Figure 1 (group of parents) and Figure 2 (group of coaches).

As presented in Figure 1, the direct effect path-way (path c’) of anger expression (β = −0.065, SE = 0.02, *t*(198) = −2.526, *p* = 0.01) had a significant and negative impact on the performance score, indicating anger as a significant predictor of performance.

The direct effects of anger expression on self-efficacy (β = −0.386, SE = 0.03, *t*(198) = −11.992, *p* < 0.001), general assertiveness distress (β = 1.068, SE = 0.07, *t*(198) = 13.847, *p* < 0.001), and general assertiveness performance (β = −0.574, SE = 0.04, *t*(198)= −13.150, *p* < 0.001), were statistically significant. These findings indicated that parents scoring higher on anger exhibited lower levels of self-efficacy, higher levels of general assertiveness distress, and lower levels of general assertiveness performance than those scoring lower on the condition. Furthermore, the direct effect of self-efficacy (β = 0.163, SE = 0.04, *t*(198) = 4.061, *p* < 0.001) was statistically and positively significant on performance score. Meanwhile, the direct effect of general assertiveness distress (β = −0.028, SE = 0.01, *t*(198) = −1.947, *p* < 0.05) was statistically and negatively significant on performance score. The direct effect of general assertiveness performance (β = 0.036, SE = 0.02, *t*(198)= 1.440, *p* = 0.14) on performance score was not statistically significant.

As presented in Figure 2, the direct effect path-way (path c’) of anger expression (β = −0.079, SE = 0.03, *t*(198) = −2.077, *p* = 0.03) had a significant and negative impact on the performance score, indicating anger as a significant predictor of performance. The direct effects of anger expression on self-efficacy (β = −0.270, SE = 0.04, *t*(198) = −5.610, *p* < 0.001), general assertiveness distress (β = 0.373, SE = 0.09, *t*(198) = 3.806, *p* < 0.01), and general assertiveness performance (β = −0.664, SE = 0.10, *t*(198) = −6.130, *p* < 0.001) were statistically significant.

These findings indicated that coaches scoring higher on anger exhibited lower levels of self-efficacy, higher levels of general assertiveness distress, and lower levels of general assertiveness performance than those scoring lower on the condition.

Furthermore, the direct effect of self-efficacy (β = 0.097, SE = 0.04, *t*(198) = 2.080, *p* < 0.05) was statistically and positively significant on performance score. The direct effect of general assertiveness distress (β = −0.126, SE = 0.03, *t*(198) = −3.448, *p* < 0.01) was statistically and negatively significant on performance score. Meanwhile, the direct effect of general assertiveness performance (β = 0.110, SE = 0.03, *t*(198) = 3.317, *p* < 0.01) was statistically and positively significant on performance score.

The indirect effects of three mediators tested simultaneously are presented in Table 3 for the groups of parents and Table 4 for the group of coaches, which contain the standardized β, indicating the intensity of the effect, and the 95% CIs, indicating the significance of the effect with a 5% probability of error (CIs that do not contain 0 are significant).

The results showed that anger expression had a significant indirect effect mediated by self-efficacy (IE = −0.063, CI = −0.112~−0.035) and general assertiveness distress (IE = −0.030, CI = −0.074~−0.006), on performance score. Thus, the relationship between anger and performance was fully mediated by self-efficacy and general assertiveness distress, with a decrement of anger expression.

The results showed that anger expression had a significant indirect effect mediated by self-efficacy (IE = −0.026, CI = −0.068~−0.003), general assertiveness distress (IE = −0.047, CI = −0.113~−0.020), and general assertiveness performance (IE = −0.073, CI = −0.156~−0.036) on performance score. Thus, the relationship between anger and performance was fully mediated by self-efficacy, general assertiveness distress, and general assertiveness performance, with a decrement of anger expression.

## 4. Discussion

According to the World Health Organization [40], youth physical and sport activity is considered of great importance to children’s general wellness. In this regard, it has numerous benefits for health, correct development, and positive character-building experience. However, an overemphasis on winning, excessive anxiety, and adverse relationships with coaches, parents, or peers can negatively influence children’s performance [20].

The present study aimed to investigate the mediating role of self-efficacy and assertiveness in the relationship between the degree of anger expression and the performance children’s score in a group of parents and coaches. We hypothesized that index anger expression would be related to performance score through the mediation of self-efficacy and general assertive behavior. Data analysis conclusively confirmed the research hypotheses.

Firstly, the comparison between parents and coaches revealed that the general assertiveness distress and the anger expression were significantly higher in parents than in coaches. Koruklu [41] showed that highly assertive individuals view a worrying situation as a challenge, while those with a low assertive level evaluate it as a threatening stressful situation. These individuals believe that they may not be able to control threats and experience a higher anxiety-provoking stimulus, looking upon many aspects of their environment with anger. This finding supports previous research that indicates parents yell most at their own child, reporting moderately frequent angry interactions during youth sport events [29,42]. Moreover, coaches had significantly higher levels of general assertiveness performance. The relationship with youth sports has a potential and positive impact on the level of assertiveness in coaches, improving also interpersonal communication [26].

The comparison between low-performance score (<60) and high-performance score (>60) highlighted that self-efficacy and general assertiveness performance were significantly higher in high performance score than in low performance score. Moreover, general assertiveness distress and anger expression were significantly lower in high performance score than in low performance score. Some parents fail to realize that their behaviors often characterized by anger may create a perfect storm, which may adversely affect the experience and performance of youth sport participants.

Youth sport situations which are characterized by verbal anger, such as parents yelling at coaches, or physical anger may be exclusively stressful to children, leading a poor performance [25]. Stirling and Kerr [43] found reduced performance effects in athletes from a range of different sports regarding their experiences of emotional abuse. Sporting success is generally determined by the performance outcome, and then the notion of “the end justifies the means” is frequently reported inside sport contexts. Clearly, a coach who seems calm and in control will have a more positive effect on the match than a coach who seems to be stressed or angry [17]. The ability to maintain emotional control to intense pressure also includes the influence of factors such as self-efficacy and self-confidence [44,45].

In addition, Spearman’s rho correlation, revealing that scores for self-efficacy were significantly and negatively associated with scores for general assertiveness distress and anger expression, confirmed the initial hypothesis (H1). Moreover, anger expression was significantly and positively associated with scores for general assertiveness distress and negatively associated with general assertiveness performance. These findings are in line with the consideration of Wolpe and Lazarus [46], whose definition of the assertiveness state includes the expression of anger and dissatisfaction. In fact, the lower the self-efficacy, the higher a more negative attitude, a sense of threat and inability to control a stressful situation. Generally, there is a positive relationship between self-efficacy and positive attitude and wrestling with the problem. On the other hand, scores of general assertiveness performance were significantly and positively associated with self-efficacy. Having a high level of self-efficacy, an individual responds more assertively in interacting with others [46].

These findings are consistent with those of previous studies [47,48], confirming a high positive correlation between self-efficacy level and assertiveness. According to the literature, assertiveness and self-efficacy, as intrapersonal dimensions to cope with environmental demands, represent important social skills which promote an individual’s well-being. Because of these considerations having a high level of self-efficacy, an individual responds more assertively in interacting with others [49,50]. Higher self-efficacy creates a sense of control through beliefs that optimistic outcomes are achievable, resulting in encouraging guides, and support for performance [51].

The last investigation of the present study—a mediation analysis—showed that in both groups, anger expression was negatively associated with performance score indirectly via self-efficacy. Specifically, we found that anger expression indirectly predicted performance score through self-efficacy. In line with the current literature on sport coaching, self-efficacy relates to the coaches’ perception of one’s ability to produce desired athletic outcome in athletes and to significantly improved sport performances [52,53,54]. Sullivan et al. [19] found that coaches with lower levels of self-efficacy had a decreased perception of athlete skills and a lower level of coaching education compared with coaches who reported higher levels of self-efficacy. Parents also play an important source of influence on their children through the transmission of values, beliefs, and expectations. In addition to providing inspirational messages, credible coaches also structure activities that bring success and improvement of performance [55].

Additionally, in both groups, anger expression was negatively associated with performance score indirectly via general assertiveness distress, while the direct effect was positive and significant. Thus, the relationship between anger and performance was fully mediated by self-efficacy and general assertiveness distress, with a decrement of anger expression. It is known that parental attitude in the sport context influences the children’s sports practice, taking on a monitoring and support role. In this regard, parents frequently react during sport events with aggressive behaviours toward coaches and referees, exhibiting negative and critical behaviours [56,57]. Horne and colleagues [58] found poor communication, wariness, and lack of collective goals between coaches and parents. Yabe et al. [24] found that parents’ dissatisfaction was associated with their lack of communication with coaches.

Finally, only in the coaches’ group was anger expression negatively associated with performance score indirectly through general assertiveness performance; however, the direct effect was negative and significant. Some research studies have found that angry reactions experienced by parents are generally caused by the perception that a coach, athlete, or other parent spectator has acted in a way inappropriate or incompetent or has engaged in behaviour that demonstrated a lack of attention. Parents have many expectations regarding the competence of coaches [59,60]. These findings highlight the significant role of coaches and parents in supporting positive sport behaviors and increasing performance levels.

Moreover, the direct effect pathway of anger had a significant and negative impact on performance score, indicating anger as a significant predictor of performance. These findings highlighted the substantial impact of anger expression on performance outcome, showing that exposure to anger is distressing for children at youth sport contexts. In conclusion, the mediation confirmed almost all research hypotheses. Specifically, mediation analyses highlighted the role of self-efficacy and general assertiveness as significant components for improvement of performance outcome, orienting individuals to have beliefs with which they can cope under pressure circumstances [61].

In interpreting the findings, the several limitations of this study should be acknowledged. First, the influence on performance was assessed only as a perception, and no definitive measures were taken. Furthermore, the study design is cross-sectional, and, therefore, no conclusions about the direction of relationships are possible. Future longitudinal studies would be helpful to further understand patterns of self-efficacy, assertiveness, and anger. Finally, this study included parents of children involved in youth sports in Italy, and it is not clear whether the findings of this study can be generalized to other countries. Future research with a large and representative sample would enrich and strengthen the evidence that are documented in the current study.

However, this study also has strengths. In this regard, it highlights an important contribution through considering and understanding the situational and interpersonal factors that influence the coach–parent relationship, impacting the level performance. This is relevant to learn about parents’ attitudes regarding the sports practice of their children. Similarly, coaches should be trained to react to parents’ attitudes. This will enhance athlete development, creating an appropriate communication and a positive climate. Youth sport is a global system, which includes coaches, referees, athletes, and parent spectators; consequently, the behavior of any member can influence the experiences of others.

Coaches should be a virtuous example for parents and athletes by respecting all members of the system and caring for the children. Equally, parents should make an effort to understand how their conduct influences the emotional well-being and performance of their children, showing respect for coaches during youth sport contexts.

## 5. Conclusions

Youth sport provides fun and regular physical activity for millions of children. The goal of each athlete is to achieve a successful performance and to minimize and control interferences. These experiences are possible through active adult involvement, especially on the part of coaches and parents. Therefore, it is important to create positive sports environments in which young athletes enjoy their experiences and are motivated to continue participating. An understanding of the nature and magnitude of this profoundly interpersonal experience can help to create relevant education programs and effective interventions aimed at reducing angry expressions and lack of communication.

## Figures and Tables

**Table 1:** Group differences in the dimensions of the study. (Student’s *t*-test, *p* < 0.05).

Dimension	Parents		Coaches		*t*	*p*	Cohen’s d
	M	SD	M	SD			
Self-efficacy	36.41	9.34	37.49	7.45	−1.27	0.202	−0.13
General assertiveness distress	66.64	23.88	62.46	14.60	2.10	0.036	0.22
General assertiveness performance	74.13	13.18	77.18	16.97	−2.01	0.045	−0.20
Anger expression	73.60	15.68	70.50	10.20	2.34	0.020	0.24

**Table 2:** Performance score differences in the dimensions of the study. (Student’s *t*-test, *p* < 0.05).

Dimension	Low Performance Score		High Performance Score		*t*	*p*	Cohen’s d
	M	SD	M	SD			
Self-efficacy	31.43	6.40	43.28	5.61	−19.53	0.001	−1.95
General assertiveness distress	76.89	14.88	50.35	14.78	17.83	0.001	1.79
General assertiveness performance	65.22	9.01	87.66	11.74	−21.18	0.001	−2.16
Anger expression	79.51	12.52	63.46	7.90	15.52	0.001	1.51

**Table 3:** Summary of indirect effects and confidence intervals of three mediators (Parents).

Mediators	Indirect Effects	SE	*p*	Boot LLCI	Boot ULCI
Anger expression → Self-efficacy → Performance score	−0.063	0.019	<0.01	−0.112	−0.035
Anger expression → General assertiveness distress → Performance score	−0.030	0.018	<0.01	−0.074	−0.006
Anger expression → General assertiveness performance → Performance score	−0.021	0.016	0.11	−0.057	0.008

**Table 4:** Summary of indirect effects and confidence intervals of three mediators (Coaches).

Mediators	Indirect Effects	SE	*p*	Boot LLCI	Boot ULCI
Anger expression → Self-efficacy → Performance score	−0.026	0.026	<0.01	−0.068	−0.003
Anger expression → General assertiveness distress → Performance score	−0.047	0.036	<0.01	−0.113	−0.020
Anger expression → General assertiveness performance → Performance score	−0.073	0.041	<0.01	−0.156	−0.036

## Data Availability

The dataset used and analyzed in the current study is available from the corresponding author upon reasonable request.
